# Prognosis of sciatica and back-related leg pain in primary care: the ATLAS cohort

**DOI:** 10.1016/j.spinee.2017.10.071

**Published:** 2018-06

**Authors:** Kika Konstantinou, Kate M. Dunn, Reuben Ogollah, Martyn Lewis, Danielle van der Windt, Elaine M. Hay, ATLAS Study Team

**Affiliations:** Arthritis Research UK Primary Care Centre, Research Institute for Primary Care & Health Sciences, Keele University, Staffordshire ST5 5BG, United Kingdom

**Keywords:** Leg pain, Low back pain, Primary care, Prognosis, Prognostic factors, Sciatica

## Abstract

**Background Context:**

Evidence is lacking on the prognosis and prognostic factors of back-related leg pain and sciatica in patients seeing their primary care physicians. This evidence could guide timely appropriate treatment and referral decisions.

**Purpose:**

The present study aims to describe the prognosis and prognostic factors in primary care patients with low back-related leg pain and sciatica.

**Study Design:**

This is a prospective cohort study.

**Patient Sample:**

The present study included adults visiting their family doctor with back-related leg pain in the United Kingdom.

**Outcome Measures:**

Information about pain, function, psychological, and clinical variables, was collected. Good outcome was defined as 30% or more reduction in disability (Roland-Morris Disability Questionnaire).

**Methods:**

Participants completed the questionnaires, underwent clinical assessments, received a magnetic resonance imaging scan, and were followed-up 12 months later. Mixed-effects logistic regression evaluated the prognostic value of six a priori defined variable sets (leg pain duration, pain intensity, neuropathic pain, psychological factors, clinical examination, and imaging variables). A combined model, including variables from all models, examined independent effects. The National Institute for Health Research funded the study. There are no conflicts of interest.

**Results:**

A total of 609 patients were included. At 12 months, 55% of patients improved in both the total sample and the sciatica group. For the whole cohort, longer leg pain duration (odds ratio [OR] 0.41; confidence interval [CI] 0.19–0.90), higher identity score (OR 0.70; CI 0.53–0.93), and patient's belief that the problem will last a long time (OR 0.27; CI 0.13–0.57) were the strongest independent prognostic factors negatively associated with improvement. These last two factors were similarly negatively associated with improvement in the sciatica subgroup.

**Conclusions:**

The present study provides new evidence on the prognosis and prognostic factors of back-related leg pain and sciatica in primary care. Just over half of patients improved at 12 months. Patient's belief of recovery timescale and number of other symptoms attributed to the pain are independent prognostic factors. These factors can be used to inform and direct decisions about timing and intensity of available therapeutic options.

## Introduction

Low back pain (LBP) is the leading cause of years lived with disability worldwide [1] and one of the most common reasons for seeking healthcare for musculoskeletal pain [2]. Approximately 60% of patients visiting primary care with LBP report back-related leg pain [3]. Some patients will have symptoms of nerve root entrapment (commonly described as sciatica), and some will have referred leg pain, which does not involve a nerve root. Both presentations are associated with worse overall outcomes compared to LBP alone [3], [4]. The course of back-related leg pain and sciatica in primary care is often conflated with that of nonspecific LBP, as most LBP cohorts include patients with and without leg symptoms [5] and initial management advice is similar for both nonspecific and sciatica presentations [6].

Given the high probability of long-standing pain and disability in nonspecific LBP, and limited potential for diagnostic information to guide clinical decision-making, much research has focused on describing prognosis and identifying prognostic factors [7], [8] which supports planning of health-care resources and can underpin appropriate management decisions. Knowledge of prognostic factors in LBP seems to have led to better treatment decision-making and improved health and cost outcomes [9]. Such evidence is scarce for patients resenting to primary care with back-related leg pain and sciatica [10], [11]. The limited, and sometimes conflicting, evidence regarding prognostic factors in patients with sciatica hampers effective targeting of available treatments [10], [11], [12], [13], [14]; hence, the current model of care is a “stepped” escalation of available interventions [15].

Systematic reviews of predominantly secondary care cohorts indicate that factors such as age, gender, smoking, occupational workload, and neurological deficits are unlikely to be associated with outcome in sciatica [10], [11], [12]. High leg pain intensity predicted surgical intervention (which is a proxy for poor outcome with conservative management) [11], although this factor was not significantly associated with outcome in recent research [16]. For sciatica in particular, authors have suggested that clinical decision-making is hampered by the lack of evidence on prognostic factors and the almost nonexistent evidence from primary care, the setting where most patients are assessed and managed [11]. Studies investigating prognosis and prognostic factors in back-related leg pain including sciatica, with a focus in primary care, are therefore needed.

The aims of this study were (1) to describe the overall prognosis, (2) better understand the associations between potentially important prognostic factors and disability, and (3) identify the strongest factors independently associated with disability.

## Methods

### Study design and participant recruitment procedures

This is a prospective cohort study including patients aged 18 years and older, visiting their family doctor (general practitioner [GP]) with symptoms of low back-related leg pain, including sciatica, of any severity and duration. The project was approved in accordance with the agreed procedure with the South Birmingham Research Ethics Committee (REC Ref. 10/H1207/82). The study protocol, baseline assessments, and patients' characteristics are reported in detail elsewhere [17], [18]. Here, we give brief details of the recruitment process. Potentially eligible patients were identified consecutively at the GP consultation and through weekly downloads of electronic records with a diagnostic code of low back-related leg pain. Patients were sent a letter with an invitation to telephone the research center to make an appointment at the initial research clinic, information about the study, and baseline questionnaires capturing sociodemographic, pain, psychological, and health variables. At the research clinic, patients underwent a standardized clinical examination by a physiotherapist with experience in assessment and management of LBP and sciatica, and eligibility was further assessed. Patients were diagnosed having sciatica or referred (nonspecific) leg pain based on the examiner's clinical opinion. In the context of the study, sciatica diagnosis is indicative of radicular pain with or without radiculopathy (nerve root involvement/compression). A reliability study nested in this cohort showed acceptable agreement on diagnosis [19]. Exclusion criteria were “red flag” symptoms, language problems, previous spinal surgery, being pregnant, serious mental and/or physical disorders, and currently receiving treatment (other than GP care) for the same problem. Consenting, eligible patients without contraindications to magnetic resonance imaging (MRI) received a scan within 2 weeks of their baseline examination (details of the scan parameters and reporting are fully described elsewhere [17]). Patients completed self-reported questionnaires at baseline, 4 months, and 12 months. Patients received evidence-based care according to current national and international guidelines on the management of LBP and sciatica, which was recorded on case report forms, and their participation in the study did not confer any specific advantages or benefits as a result. The results of the MRI scan were not included in initial diagnosis and decisions about patient care; this reflects normal practice in primary care settings. However, the MRI findings for each patient were correlated with the clinical presentation for the MRI variable of “presence/absence of nerve root compression.”

### Primary outcome measure

The Roland-Morris Disability Questionnaire (RMDQ) leg pain version (23 items scored from 0 to 23, with higher scores indicating higher disability) was the primary outcome measure [20]. Improvement was defined as 30% or more decrease in an individual's RMDQ score between baseline and follow-up [21].

### Potential prognostic factors

Prognostic factors to be examined were a priori selected based on evidence of their association with long-term outcome, building on exploratory evidence from existing studies in LBP and sciatica, and expertise within the study team. The self-reported and clinical assessment variables investigated in the study are summarized in Table 1.

**Table 1:** List of the preselected sets of variables used in the analysis

Variable set	Domain	Measure
1	Duration of pain	Current episode of leg pain: Less than 6 wk, between 6 and 12 wk, more than 3 mo
2	Pain intensity	Taking the highest of either back or leg pain using the mean of three 0 to 10 numerical rating scales for “least,” “usual,” and “current” pain over the previous 2 wk [22]
3	Neuropathic pain features	Using the Leeds Assessment Neuropathic Symptoms and Signs (S-LANSS); with a score of 12 or more indicating possible neuropathic pain [23]
4	Psychological perceptions	Pain self-efficacy: Measured with the Pain Self-Efficacy Questionnaire (PSEQ); with scores from 0 to 60; higher scores reflect stronger self-efficacy beliefs [24] Identity; Symptom attribution to the condition [25] from a list of seven potential symptoms: back pain, leg pain, unable to sit comfortably, fatigue, stiff joints, sleep difficulties, loss of strength. The score is the sum of symptoms experienced. The list of the seven potential symptoms was chosen by the research team. Timeline; illness/condition duration: “My back and/or leg problem will last for a long time”* Personal control: How much influence a patient has over illness/condition; “There is a lot which I can do to control my back and/or leg symptoms”* Depression: Measured using the Hospital Anxiety and Depression scale (HADs); with scores from 0 to 21, higher scores indicate higher levels of depressive symptoms [26]
5	Clinical examination	Pins and needles or numbness in leg(s) as reported by the patient. Leg pain increased by coughing/laughing/straining. Worse pain, either in low back or leg. Neurological examination variables: –Myotomal strength†: Defined as normal (5 on Oxford scale)/abnormal (0, 1, 2, 3, or 4 on Oxford scale) –Reflex (tendon): Defined as normal, slightly reduced, significantly reduced/absent –Sensation‡ (in leg(s)) Defined as normal/abnormal –Neural tension test findings: Defined as abnormal if any neural tension test is abnormal (ie, straight leg raise, femoral stretch, slump)
6	Imaging (MRI) examination	MRI findings: Defined as normal when no evidence of nerve root compression correlating with clinical symptoms, or indicative of nerve root compression if there was evidence of clear or possible nerve root compression for any reason. All MRIs were scored by the same experienced consultant radiologist who had no knowledge of the specific patient presentation other than “LBP with leg pain.”

LBP, low back pain; MRI, magnetic resonance imaging.

* Timeline and Personal control are measured on a Likert scale: Strongly disagree—Disagree—Neither agree or disagree—Agree—Strongly agree. For the purposes of the analysis, it was dichotomized (agree [*agree, strongly agree*] vs. disagree [*strongly disagree, disagree, neither agree, or disagree*]).

† Muscle strength tested according to the Oxford scale, where 0, no movement; 1, flicker of movement; 2, through full range actively with gravity counterbalanced; 3, through full range actively against gravity; 4, through full range actively against some resistance; 5, through full range actively against strong resistance.

‡ Sensation was tested with a pin (neurotip).

### Treatment pathways

Participants were managed according to one of three care pathways: (1) up to two physiotherapy sessions for those patients with improving or mild symptoms, (2) a course of physiotherapy treatment (three and more) for those patients with more troublesome pain, and (3) referral to secondary care; most patients in this pathway initially received a course of physiotherapy treatment. Secondary care options included referral to pain clinic for consideration of specialist analgesia review and/or injections, or to spinal orthopaedics for consideration of surgery and/or injections, or to chronic pain management services. Choice of pathway was based on clinician's judgment and patients' preferences.

### Data analysis

The following analyses were conducted for the whole cohort, and separately for the subgroup clinically diagnosed with sciatica.

### Overall prognosis

Descriptive analysis was performed to describe the course of patients' disability and pain using mean (standard deviation [SD]) scores for RMDQ and pain intensity (LBP and leg pain) at baseline and each month, with 4 and 12 months being the main follow-up points. The percentage of patients defined as improved on the RMDQ was calculated.

### Analysis of prognostic factors

A mixed-effects logistic regression model, which allows all available outcome data at all three time points to be used, accounts for autocorrelation due to repeated measures, and gives valid inferences when data are assumed missing at random, was used to estimate odds ratios (OR) and 95% confidence interval (CI) for the association between each of the potential prognostic factors and the binary outcome of improvement in disability. The model included an interaction term between each predictor and time to obtain estimates (and 95% CI) for each point of follow-up (4 and 12 months).

Univariable associations were described, followed by a series of models evaluating the prognostic value of the six sets of variables relating to the six domains described in Table 1.

Previous research and expertise has highlighted these six domains as important, although it is unclear which factors specifically are most strongly associated with outcome within and between domains. Univariable analysis was first used to examine associations between each factor and outcome. Each model was then adjusted for (1) variables in the model only (for models with more than 1 factor); (2) age, gender, body mass index, smoking, and comorbidities; and (3) care pathways. Correlations between individual prognostic factors were investigated using bivariate associations and variance inflation factor, and if this was the case (variance inflation factor≥5), then only one of the variables (with higher OR) was included in analyses.

Finally, a combined model comprising all variables in the six models was fitted to identify the strongest factors from the six domains predicting long-term outcome. This was performed with a backwards approach by removing nonsignificant variables from the model one by one, until remaining variables had p<.05 (using the likelihood ratio test).

As a sensitivity analysis for addressing missing data, multiple imputation was employed by combining results from 40 multiply imputed datasets. Additional sensitivity analysis using linear mixed model was also performed using numerical RMDQ, with adjustment for baseline RMDQ score as the outcome, as opposed to the binary classification. In a further sensitivity analysis, a combined model using the subsample of participants with sciatica and confirmed nerve root compression on MRI was fitted.

In this study, the total number of factors considered complied with the rule of at least 10 events per variable in the logistic regression analysis [27].

### Assessment of nonresponse

For primary follow-up time points (4 and 12 months), we compared the key patient baseline characteristics (age, gender, area deprivation, pain intensity, leg pain duration, and RMDQ scores) for those followed up and those lost to follow-up.

Data were analyzed using Stata 13 (StataCorp. 2013, College Station, TX, USA).

## Results

### Study sample

Six hundred nine patients were included in the study. Response rates were 402 (66.0%) at 4 months and 450 (73.9%) at 12 months; 74.2% (n=452) were clinically diagnosed as having sciatica. The Figure presents the study flowchart. Baseline characteristics of the study population are presented in Table 2. Forty-seven percent, 41.5%, and 11.5% of patients followed care pathways (1), (2), and (3), respectively. Fourteen patients reported that they underwent spinal surgery and 21 had spinal injections.

**Fig. 1:**
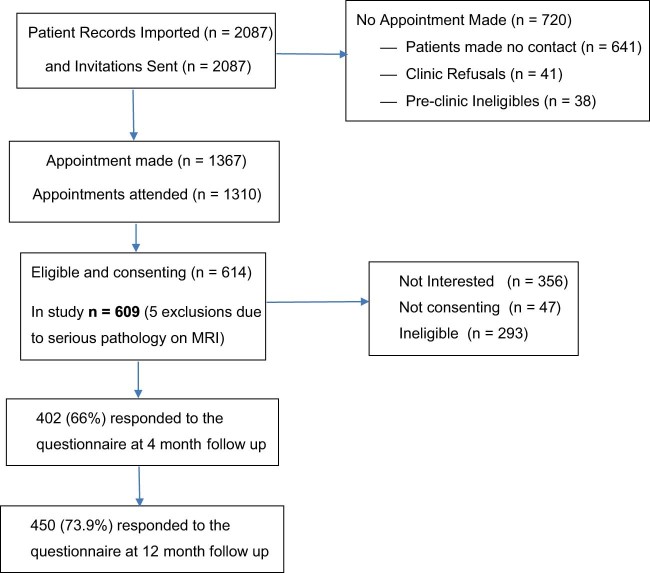
Flowchart of study population.

**Table 2:** Baseline characteristics of participants for the whole group and for the sciatica and referred leg pain subgroups

	All participants n=609	Sciatica subgroup n=452	Referred leg pain n=157
Sociodemographics (Denominator*)			
Age (y) (609), mean (SD)	50.2 (13.9)	50.4 (14.0)	49.4 (13.7)
Gender (609), Female	381 (62.6)	276 (61.1)	105 (66.9)
BMI (609), mean (SD)	29.9 (7.0)	29.9 (6.3)	30.0 (8.7)
Current smoker (609)	194 (31.9)	151 (33.4)	43 (27.4)
Comorbidities† (609)			
None	371 (60.9)	277 (61.3)	94 (59.9)
One other health problem	158 (25.9)	122 (27.0)	36 (22.9)
Two or more other health problems	80 (13.1)	53 (11.7)	27 (17.2)
**Pain and function**			
RMDQ disability score (0–23) (609), mean (SD)	12.7 (5.7)	12.9 (5.7)	12.0 (5.7)
Back pain intensity (mean of 3 NRS) (609), mean (SD)	5.6 (2.2)	5.6 (2.2)	5.4 (2.1)
Leg pain intensity (mean of 3 NRS) (608), mean (SD)	5.2 (2.4)	5.6 (2.3)	4.1 (2.3)
Duration of symptoms			
Back pain (607)			
<6 wk	218 (35.9)	174 (38.6)	44 (28.2)
6–12 wk	126 (20.8)	96 (21.3)	30 (19.2)
3–6 mo	92 (15.2)	75 (16.6)	17 (10.9)
Over 6 mo	171 (28.2)	106 (23.5)	65 (41.7)
Leg pain (583)			
<6 wk	251 (43.1)	192 (44.2)	59 (39.6)
6–12 wk	120 (20.6)	94 (21.7)	26 (17.5)
3–6 mo	84(14.4)	62 (14.3)	22 (14.8)
Over 6 mo	128 (22.0)	86 (19.8)	42 (28.0)
Leg pain is worse (609)	280 (46.0)	252 (55.8)	28 (17.8)
S-LANSS (possible neuropathic pain) (607)	332 (54.8)	232(51.6)	61 (39.0)
**Psychological measures and perceptions**			
HADs depression subscale (continuous score) (609), mean (SD)	6.4 (4.0)	6.3 (4.0)	6.4 (4.0)
HADs depression subscale: categorized (609)			
Normal (0–7)	392 (64.4)	295 (65.3)	97 (61.8)
Possible case (8–10)	119 (19.5)	82 (18.1)	37 (23.4)
Probable case (≥11)	98 (16.1)	75 (16.6)	23 (14.7)
Pain self-efficacy score‡ (593), mean (SD)	34.1 (14.6)	33.3 (14.7)	36.6 (13.9)
Illness perception			
–Identity score§ (609), mean (SD)	5.9 (1.3)	5.9 (1.3)	5.9 (1.2)
–Timeline (“back/leg pain will last forever” [agree/strongly agree]) (609)	345 (56.7)	249 (55.1)	96 (61.2)
–Personal control (“what I do can determine whether back/leg pain gets better/worse” [agree/strongly agree]) (605)	367 (60.7)	277 (61.8)	90 (57.3)
**Clinical assessment**			
Pins and needles and/or numbness (patient reports having these symptoms) (609)	382 (62.7)	316 (69.9)	66 (42.0)
Cough, sneeze or strain (patient reports increased leg pain with cough/sneeze/strain) (609)	129 (21.2)	120 (26.6)	9 (5.7)
Leg pain is worse than back pain (patient report) (609)	280 (46.0)	252 (55.8)	28 (17.8)
Myotomal change (as per Oxford scale) (608)			
5/5 (None)	503 (82.7)	347 (76.8)	156 (100)
4/5	92 (15.1)	92 (20.4)	0 (0.0)
0/5 or 1/5 or 2/5 or 3/5	13 (2.1)	13 (2.9)	0 (0.0)
Reflex change (at ankle or patella) (609)			
None	490 (80.5)	341 (75.4)	149 (94.9)
Slightly reduced	30 (4.9)	30 (6.6)	0 (0.0)
Significantly reduced	22 (3.6)	19 (4.2)	3 (1.9)
Absent	67 (11.0)	62 (13.7)	5 (3.2)
Sensory change (as examined using a pin) (609)			
None	356 (58.5)	226 (50.0)	130 (82.8)
Reduced pin/prick	201 (33.0)	175 (38.7)	26 (16.6)
Loss to pin/prick	52 (8.5)	51 (11.3)	1 (0.6)
Neural tension test positive (any) (609)	335 (55.0)	324 (71.7)	11 (7.0)
MRI (554)			
Findings of nerve root compression	297 (53.6)	252 (60.7)	45 (32.4)

BMI, body mass index; HADs, Hospital Anxiety and Depression scale; NRS, Numerical Rating Scale; RMDQ, Roland-Morris Disability Questionnaire; SD, standard deviation; S-LANSS, self-report Leeds Assessment Neuropathic Symptoms and Signs.

Note: All figures are frequencies and percentages (%), unless stated otherwise as mean and SD.

* The number of participants for each variable is shown in parentheses—the denominator varies for some participants due to missing data or not applicable case.

† The health problems included chest problems, heart problems, raised blood pressure, diabetes, and circulation problems in the leg.

‡ Ten-item scale, score range=0–60—higher scores reflect stronger self-efficacy beliefs.

§ Sum of scores on seven symptoms—higher scores represent strongly held beliefs about number of symptoms attributed to the illness**.**

Participants who did not respond to the 12-month questionnaire were more often male, younger, and had higher baseline disability score compared with responders (see Table S1 for details).

### Prognosis of low back-related leg pain and sciatica

Overall, 55.0% of the cohort reported improvement at 12 months, both in the sciatica and in the referred leg pain subgroups. At baseline, mean disability was 12.6 (SD 5.7); this had decreased to means of 8.2 (6.7) and 7.8 (7.0) at 4 and 12 months, respectively. Baseline mean back pain intensity was 5.6 (2.2); this decreased to 3.4 (2.6) and 3.3 (2.7) at 4 and 12 months, respectively. For leg pain, mean baseline pain intensity was 5.2 (2.4), falling to 2.8 (2.9) and 2.4 (2.7) at 4 and 12 months, respectively.

### Prognostic factors associated with long-term changes in disability

Table 3 shows all univariable associations between baseline variables and disability. Multivariable analysis with sequential adjustment for other variables in the models, demographics, and care pathways identified factors significantly associated with outcome in each predefined domain or variable set (presented in Table 4, Table 5). Longer leg pain duration (OR 0.30, 95% CI 0.13–0.66), higher pain intensity (OR 0.84, CI 0.71–0.99), higher identity score (OR 0.68, CI 0.50–0.93), and patient's belief that the problem will last a long time (OR 0.29, CI 0.14–0.60) were negatively associated with improvement, whereas having myotomal weakness (OR 4.56, CI 1.69–12.33) was positively associated with improvement. With the exception of pain intensity, the same prognostic factors were significant in the sciatica subgroup.

**Table 3:** Univariable associations between baseline variables and improvement in the RMDQ at 4 and 12 months for the whole group and sciatica subgroup based on mixed-effects logistic regression model (statistically significant values in bold)

Improved vs. not improved: reference category in parentheses	All participants		Sciatica subgroup	
	4 mo (n=402)	12 mo (n=450)	4 mo (n=308)	12 mo (n=338)
	OR (95% CI)	OR (95% CI)	OR (95% CI)	OR (95% CI)
**Pain variables (Sets 1, 2, 3)**				
Duration of leg pain (<6 wk)				
6–12 wk	0.73 (0.28–1.93)	1.02 (0.39–2.61)	0.55 (0.17–1.79)	1.10 (0.35–3.46)
Over 3 mo	**0.16 (0.07–0.38)**	**0.23 (0.10–0.52)**	**0.09 (0.03–0.29)**	**0.19 (0.07–0.56)**
Pain intensity (cont)	**0.66 (0.55–0.79)**	**0.74 (0.63–0.89)**	**0.62 (0.49–0.79)**	**0.73 (0.58–0.91)**
S-LANSS: possible neuropathic pain (No)	**0.31 (0.14–0.65)**	**0.46 (0.23–0.93)**	**0.26 (0.10–0.66)**	**0.37 (0.15–0.92)**
**Psychological measures and perceptions (Set 4)**				
Pain self-efficacy	**1.05 (1.02–1.08)**	**1.04 (1.01–1.06)**	**1.04 (1.01–1.08)**	**1.04 (1.01–1.08)**
Illness perception				
–Identity*	**0.54 (0.40–0.73)**	**0.52 (0.38–0.69)**	**0.48 (0.33–0.69)**	**0.48 (0.33–0.70)**
–Timeline†	**0.33 (0.16–0.69)**	**0.20 (0.09–0.41)**	**0.29 (0.11–0.73)**	**0.12 (0.05–0.33)**
–Personal control†	1.40 (0.66**–**2.96)	1.55 (0.76**–**3.18)	1.37 (0.54**–**3.51)	1.61 (0.65**–**4.00)
HADs depression (cont)	**0.82 (0.75–0.91)**	**0.86 (0.78–0.94)**	**0.82 (0.73–0.93)**	**0.82 (0.73–0.93)**
**Clinical assessment and imaging (Set 5, 6)**				
Reporting pins and needles and/or numbness (No)	0.47 (0.22**–**1.02)	0.86 (0.42**–**1.76)	0.76 (0.30**–**1.93)	1.45 (0.59**–**3.58)
Increased leg pain with cough/sneeze/strain (No)	0.74 (0.29**–**1.87)	0.82 (0.34**–**1.94)	0.94 (0.32**–**2.79)	0.89 (0.32**–**2.47)
What is worse (Back pain)	1.58 (0⋅76**–**3.27)	1.13 (0.56**–**2.27)	3.15 (1.22**–**8.10)	1.95 (0.79**–**4.78)
Myotomes (No weakness [normal])	1.66 (0.64**–**4.29)	**2.62 (1.02–6.69)**	2.08 (0.70**–**6.16)	**3.22 (1.10–9.46)**
Reflex (Normal)				
Slightly reduced	0.19 (0.03**–**1.17)	0.72 (0.13**–**3.91)	0.18 (0.02**–**1.32)	0.69 (0.12**–**4.59)
Absent or significantly reduced	0.58 (0.20**–**1.68)	0.46 (0.17**–**1.30)	0.67 (0.19**–**2.32)	0.43 (0.13**–**1.44)
Sensation (Normal)	0.54 (0.25**–**1.13)	0.57 (0.28**–**1.17)	0.61 (0.24**–**1.53)	0.46 (0.19**–**1.12)
Neural tension test (Normal)	1.04 (0.50**–**2.16)	0.92 (0.46**–**1.85)	1.58 (0.59**–**4.23)	1.02 (0.39**–**2.68)
MRI finding: Nerve root compression (No)	1.07 (0.50**–**2.29)	1.06 (0.51**–**2.18)	1.00 (0.37**–**2.68)	1.08 (0.41**–**2.82)

CI, confidence interval; HADs, Hospital Anxiety and Depression scale; MRI, magnetic resonance imaging; OR, odds ratio; RMDQ, Roland-Morris Disability Questionnaire; S-LANSS, self-report Leeds Assessment Neuropathic Symptoms and Signs.

* Symptoms that the patient sees as part of the illness (0–7).

† Timeline and personal control are measured on a Likert scale: Strongly disagree—Disagree—Neither agree or disagree—Agree—Strongly agree. For the purposes of the analysis, it was dichotomized (agree [*agree, strongly agree*] vs. disagree [*strongly disagree, disagree, neither agree, or disagree*]). The reference for the analysis is “Strongly disagree/disagree/neither.”

**Table 4:** Association of six preselected set of variables (models 1 to 6) with improvement in RMDQ at 4 and 12 months based on mixed-effects logistic regression model for the whole group

Variables in the model (Reference category)	4 mo (n=402)			12 mo (n=450)		
	Adjusted for all the variables in the model	Adjusted for the variables in the model and demographics*	Adjusted for all the variables in the model, demographics, and care pathways	Adjusted for only variables in the model	Adjusted for the variables in the model and demographics*	Adjusted for all the variables in the model, demographics, and care pathways
	OR (95% CI)	OR (95% CI)	OR (95% CI)	OR (95% CI)	OR (95% CI)	OR (95% CI)
**Model 1**						
Duration of leg pain (<6 wk)						
6–12 wk	0.73 (0.28–1.93)	0.67 (0.26–1.73)	0.81 (0.31–2.12)	1.02 (0.39–2.61)	0.87 (0.35–2.19)	0.92 (0.3–2.31)
Over 3 mo	**0.16 (0.07**–**0.38)**	**0.22 (0.09**–**0.51)**	**0.26 (0.11**–**0.61)**	**0.23 (0.10**–**0.52)**	**0.29 (0.13**–**0.63)**	**0.30 (0.13**–**0.66)**
**Model 2**						
Pain intensity (cont)	**0.66 (0.55**–**0.79)**	**0.71 (0.60**–**0.86)**	**0.76 (0.63**–**0.91)**	**0.75 (0.63**–**0.89)**	**0.82 (0.69**–**0.97)**	**0.84 (0.71**–**0.99)**
**Model 3**						
S-LANSS: possible neuropathic origin (No)	**0.31 (0.14**–**0.65)**	**0.37 (0.18**–**0.77)**	**0.44 (0.21**–**0.91)**	**0.46 (0.23**–**0.93)**	0.60 (0.30–1.19)	0.65 (0.33–1.29)
**Model 4**						
Pain self-efficacy	1.03 (0.99–1.06)	1.02 (0.99–1.06)	1.02 (0.99–1.05)	1.00 (0.97–1.03)	0.99 (0.96–1.02)	0.99 (0.96–1.03)
Illness perception						
–Identity	**0.64 (0.46**–**0.88)**	0.74 (0.54–1.00)	0.77 (0.56–1.05)	**0.58 (0.42**–**0.80)**	**0.67 (0.49**–**0.91)**	**0.68 (0.50**–**0.93)**
–Timeline	0.52 (0.25–1.11)	0.49 (0.23–1.01)	0.55 (0.26–1.16)	**0.31 (0.15**–**0.65)**	**0.28 (0.13**–**0.59)**	**0.29 (0.14**–**0.60)**
–Personal control	1.49 (0.69–3.19)	1.57 (0.75–3.27)	1.48 (0.70–3.13)	1.52 (0.73–3.17)	1.61 (0.79–3.30)	1.60 (0.78–3.29)
HAD depression (cont)	0.95 (0.84–1.08)	0.96 (0.84–1.08)	0.96 (0.84–1.09)	0.96 (0.85–1.08)	0.96 (0.85–1.08)	0.96 (0.85–1.09)
**Model 5**						
Reporting pins and needles and/or numbness (No)	0.57 (0.26–1.26)	0.61 (0.28–1.33)	0.66 (0.30–1.44)	1.02 (0.48–2.17)	1.23 (0.59–2.57)	1.32 (0.63–2.76)
Increased leg pain with cough/sneeze/strain (No)	0.78 (0.29–2.11)	0.62 (0.24–1.64)	0.82 (0.30–2.19)	0.83 (0.33–2.09)	0.71 (0.29–1.74)	0.77 (0.31–1.88)
What is worse (Back pain)	1.65 (0.74–3.68)	1.74 (0.80–3.78)	1.72 (0.79–3.74)	1.18 (0.55–2.53)	1.24 (0.60–2.59)	1.28 (0.61–2.66)
Myotomes (No weakness [normal])	2.61 (0.92–7.41)	2.69 (0.98–7.40)	**3.47 (1.22**–**9.82)**	**3.92 (1.42**–**10.83)**	**4.10 (1.53**–**11.00)**	**4.56 (1.69**–**12.33)**
Reflex (Normal)						
Slightly reduced	**0.14 (0.02**–**0.91)**	0.16 (0.03–1.00)	0.16 (0.03–1.01)	0.52 (0.09–2.95)	0.57 (0.10–3.07)	0.58 (0.11–3.11)
Absent or significantly reduced	0.53 (0.18–1.58)	0.47 (0.16–1.35)	0.53 (0.18–1.54)	0.41 (0.15–1.17)	0.35 (0.13–0.98)	0.38 (0.14–1.05)
Sensation (Normal)	0.53 (0.24–1.16)	0.66 (0.31–1.42)	0.74 (0.34–1.59)	0.49 (0.23–1.05)	0.65 (0.31–1.36)	0.67 (0.33–1.40)
Neural tension test (Normal)	1.04 (0.46–2.36)	0.95 (0.43–2.09)	1.01 (0.45–2.25)	0.88 (0.40–1.96)	0.75 (0.35–1.63)	0.80 (0.37–1.73)
**Model 6**						
MRI finding: Nerve root compression (No)	1.07 (0.50–2.29)	0.95 (0.45–2.00)	1.30 (0.60–2.80)	1.06 (0.51–2.18)	0.95 (0.47–1.93)	1.05 (0.51–2.15)

CI, confidence interval; HADs, Hospital Anxiety and Depression scale; MRI, magnetic resonance imaging; OR, odds ratio; RMDQ, Roland-Morris Disability Questionnaire; S-LANSS, self-report Leeds Assessment Neuropathic Symptoms and Signs.

* Adjusted for age, gender, body mass index, smoking, and comorbidities.

**Table 5:** Association of six preselected set of variables (models 1 to 6) with improvement in RMDQ at 4 and 12 months based on mixed-effects logistic regression model for the sciatica subgroup

Variables in the model (Reference category)	4 mo (n=308)			12 mo (338)		
	Adjusted for all the variables in the model	Adjusted for the variables in the model and demographics*	Adjusted for all the variables in the model, demographics, and care pathways	Adjusted for only variables in the model	Adjusted for the variables in the model and demographics*	Adjusted for all the variables in the model, demographics, and care pathways
	OR (95% CI)	OR (95% CI)	OR (95% CI)	OR (95% CI)	OR (95% CI)	OR (95% CI)
**Model 1**						
Duration of leg pain (<6 wk)						
6–12 wk	0.55 (0.17–1.79)	0.50 (0.16–1.60)	0.65 (0.19–2.19)	1.10 (0.35–3.46)	0.96 (0.31–2.92)	1.01 (0.31–3.22)
Over 3 mo	**0.09 (0.03–0.28)**	**0.14 (0.05–0.41)**	**0.16 (0.05–0.50)**	**0.19 (0.07–0.56)**	**0.26 (0.09–0.73)**	**0.23 (0.08–0.69)**
**Model 2**						
Pain intensity (cont)	**0.62 (0.49–0.79)**	0.69 (0.54–0.87)	**0.75 (0.59–0.95)**	**0.73 (0.58–0.91)**	**0.78 (0.63–0.98)**	0.81 (0.64–1.01)
**Model 3**						
S-LANSS: possible neuropathic pain (No)	**0.26 (0.10–0.66)**	**0.32 (0.13–0.81)**	**0.37 (0.14–0.96)**	**0.37 (0.15–0.92)**	0.50 (0.21–1.19)	0.52 (0.21–1.26)
**Model 4**						
Pain self-efficacy	1.01 (0.97–1.04)	1.01 (0.97–1.05)	1.00 (0.9–1.04)	0.99 (0.96–1.04)	0.99 (0.96–1.04)	0.99 (0.95–1.03)
Illness perception						
–Identity	**0.54 (0.36–0.80)**	**0.63 (0.43–0.91)**	**0.65 (0.44–0.96)**	**0.56 (0.38–0.83)**	**0.64 (0.44–0.94)**	**0.64 (0.43–0.95)**
–Timeline	0.48 (0.19–1.22)	0.49 (0.20–1.21)	0.58 (0.22–1.51)	**0.20 (0.08–0.53)**	**0.22 (0.08–0.55)**	**0.21 (0.08–0.54)**
–Personal control	1.53 (0.59–3.92)	1.62 (0.65–4.02)	1.46 (0.56–3.82)	1.43 (0.57–3.62)	1.50 (0.62–3.63)	1.51 (0.60–3.81)
HADs depression (cont)	0.94 (0.80–1.10)	0.94 (0.80–1.10)	0.94 (0.80–1.11)	0.93 (0.80–1.08)	0.93 (0.81–1.08)	0.93 (0.78–1.09)
**Model 5**						
Reporting pins and needles and/or numbness (No)	0.47 (0.16–1.34)	0.57 (0.20–1.60)	0.62 (0.21–1.84)	0.93 (0.43–2.54)	1.20 (0.45–3.19)	1.37 (0.50–3.74)
Increased leg pain with cough/sneeze/strain (No)	0.87 (0.28–2.77)	0.56 (0.18–1.74)	0.74 (0.23–2.45)	0.83 (0.29–2.43)	0.66 (0.23–1.91)	0.68 (0.23–2.00)
What is worse (Back pain)	**3.00 (1.09–8.25)**	**3.07 (1.15–8.21)**	**3.10 (1.12–8.62)**	2.01 (0.77**–**5.26)	1.98 (0.78**–**5.00)	2.12 (0.85**–**5.73)
Myotomes (No weakness [normal])	2.75 (0.86**–**8.77)	2.92 (0.94**–**9.09)	**4.31 (1.27–14.63)**	**4.57 (1.45–14.40)**	**4.57 (1.51–13.82)**	**5.62 (1.76–17.92)**
Reflex (Normal)						
Slightly reduced	**0.11 (0.01–0.92)**	0.12 (0.01**–**0.99)	0.12 (0.01**–**1.07)	0.46 (0.07**–**3.19)	0.51 (0.08**–**3.34)	0.56 (0.08**–**3.79)
Absent or significantly reduced	0.56(0.16**–**2.01)	0.45 (0.13**–**1.58)	0.48 (0.13**–**1.83)	0.35 (0.10**–**1.21)	0.30 (0.09**–**1.02)	0.35 (0.10**–**1.20)
Sensation (Normal)	0.63 (0.24**–**1.65)	0.82 (0.32**–**2.07)	1.03 (0.39**–**2.72)	0.40 (0.16**–**1.03)	0.53 (0.22**–**1.30)	0.56 (0.23**–**1.41)
Neural tension test (Normal (negative)	1.22 (0.43**–**3.46)	1.13 (0.41**–**3.12)	1.28 (0.44**–**3.74)	0.84 (0.30**–**2.35)	0.68 (0.25**–**1.87)	0.81 (0.29**–**2.27)
**Model 6**						
MRI finding: Nerve root compression (No)	1.00 (0.37**–**2.68)	0.71 (0.27**–**1.88)	1.08 (0.38**–**3.05)	1.08 (0.42**–**2.82)	0.82 (0.32**–**2.09)	0.89 (0.33**–**2.39)

CI, confidence interval; HADs, Hospital Anxiety and Depression scale; MRI, magnetic resonance imaging; OR, odds ratio; RMDQ, Roland-Morris Disability Questionnaire; S-LANSS, self-report Leeds Assessment Neuropathic Symptoms and Signs.

* Adjusted for age, gender, body mass index, smoking, and comorbidities.

Adjustment for demographics and care pathways did not have a large impact on associations for most variables for all domains (Table 4, Table 5).

### Independent prognostic factors associated with long-term changes in disability

For the whole cohort, the combined multivariable model incorporating all variables from the six sets or domains showed that longer leg pain duration (OR 0.41, CI 0.19–0.90), higher identity score (OR 0.70, CI 0.53–0.93), and patient's belief that the problem will last a long time (OR 0.27, CI 0.13–0.57) were the strongest independent prognostic factors negatively associated with improvement. These last two factors were similarly negatively associated with improvement in the sciatica subgroup (Table 6). The sensitivity analyses using multiple imputation and continuous RMDQ scores as the outcome produced very similar results (data not presented). The results from the sensitivity analysis using the subsample with sciatica and corroborative MRI findings showed that “identity” remained independently associated with outcome (see Table S2).

**Table 6:** Multivariable associations between baseline characteristics and improvement in the RMDQ for the whole group and the sciatica group, combining all the six preselected set of variables

Variables in the final model (Reference category)	4 mo			12 mo		
	Adjusted for all the variables in the final model	Adjusted for the variables in the model and demographics*	Adjusted for all the variables in the model, demographics, and care pathways	Adjusted for all the variables in the final model	Adjusted for the variables in the model and demographics*	Adjusted for all the variables in the model, demographics, and care pathways
	OR (95% CI)	OR (95% CI)	OR (95% CI)	OR (95% CI)	OR (95% CI)	OR (95% CI)
**Whole group**						
Duration of leg pain (>6 wk)						
6–12 wk	0.79 (0.30–2.10)	0.75 (0.29–1.94)	0.85 (0.32–2.24)	1.11 (0.43–2.84)	1.03 (0.41–2.56)	1.04 (0.41–2.62)
Over 3 mo	0.23 (0.10–0.55)	0.28 (0.12–0.64)	0.31 (0.13–0.72)	0.36 (0.16–0.79)	0.42 (0.19–0.92)	0.41 (0.19–0.90)
–Timeline	—	—	—	0.31 (0.15–0.64)	0.27 (0.13–0.56)	0.27 (0.13–0.57)
Pain intensity (cont)	0.75 (0.63–0.91)	0.80 (0.67–0.96)	0.81 (0.67–0.97)	—	—	—
–Identity	0.66 (0.49–0.89)	0.73 (0.54–0.97)	0.74 (0.54–0.99)	0.59 (0.44–0.79)	0.69 (0.52–0.91)	0.70 (0.53–0.93)
**Sciatica subgroup**						
Duration of leg pain (>6 wk)						
6–12 wk	0.58 (0.18–1.86)	0.56 (0.18–1.76)	0.66 (0.20–2.20)	1.30 (0.41–4.10)	1.27 (0.41–3.82)	1.29 (0.40–4.19)
Over 3 mo	0.15 (0.05–0.46)	0.19 (0.07–0.57)	0.20 (0.06–0.63)	0.31 (0.11–0.88)	0.41 (0.15–1.11)	0.34 (0.11–1.01)
–Timeline	—	—	—	0.21 (0.08–0.55)	0.22 (0.09–0.56)	0.21 (0.08–0.56)
Pain intensity (cont)	0.70 (0.54–0.89)	0.75 (0.59–0.96)	0.79 (0.62–1.02)	—	—	—
–Identity	0.63 (0.43–0.90)	0.69 (0.49–0.99)	0.74 (0.51–1.07)	0.57 (0.39–0.81)	0.64 (0.45–0.91)	0.65 (0.45–0.94)
What is worse (back pain)	3.47 (1.32–9.11)	3.15 (1.22–8.12)	3.37 (1.25–9.08)	—	—	—

CI, confidence interval; OR, odds ratio; RMDQ, Roland-Morris Disability Questionnaire.

Note: Only the variables that were statistically significant in the final combined model are presented.

* Adjusted for age, gender, body mass index, smoking, and comorbidities.

## Discussion

To our knowledge, this is the first comprehensive study to describe prognosis and prognostic factors in patients seeking care in primary care for back-related leg pain, including sciatica, of any duration and severity. The prognosis of low back-related leg pain is similar in those with and without a clinical diagnosis of sciatica, with 55% meeting the study's criterion for improvement in disability. The improvements in disability from baseline in our cohort were similar, but mostly higher (mean change score; 4.8), compared with some LBP cohorts (UK) with or without leg pain, receiving primary care including physiotherapy interventions ((4.3) [28], (2.4) [29]). The percentage of patients with sciatica reporting improvement (55%) at 1 year is within the range of reports from secondary care populations, irrespective of outcome definition (range 32%–65%) [13], [14], [30].

In this study, we set out to specifically investigate prognostic factors thought to be associated with long-term outcome in low back-related leg pain and in sciatica and also examine their independent effect.

The factors associated with improvement in disability in this cohort were shorter pain duration, lower leg pain intensity, fewer other symptoms associated with the back and leg pain (lower identity score), patient's belief that the problem will be short-lived, and initially having myotomal weakness. Symptom duration and pain levels are similarly reported to be associated with better outcomes in nonspecific LBP [31], [32].

For the sciatica subgroup, pain intensity was not statistically significant after adjustment for care pathways, which perhaps indicates that treatment modifies its effect (although the strength of association fell by only 0.03). This contrasts with the current secondary care literature which points to leg pain severity in sciatica as likely associated with subsequent surgery (proxy of poor outcome with conservative management) [11]. More recently, Suri et al. [16] also did not confirm leg pain intensity as being associated with disability in conservatively treated sciatica patients.

Depression was not found to be a significant factor when included with other psychological variables in the model. This is consistent with results from other studies, where factors of “timeline” and “identity” are independent and stronger prognostic factors in nonspecific LBP when compared to depression [33]. In our cohort, the expectation of getting better soon was only relevant in the long term, which may be indicative of the interplay between natural course and initial treatment effect. “Identity” was a significant prognostic factor for the sciatica subgroup, across both time points, with decreased odds of improvement for each increase in score. The “identity” score was the sum of symptoms including sleep disturbance, fatigue, unable to sit comfortably, all of which are often reported by patients with back and leg pain, and sciatica, and may be reasonably considered as an overall indication of severity or impact of symptoms. However, both these characteristics may well be influenced by patients' behavior and psychological profile, such as a pessimistic outlook for example.

We found that having myotomal weakness at baseline was associated with improvement in disability at 12 months. All patients with myotomal weakness had additional neurologic signs (ie, reflex or sensory change). One recent study in secondary care [14] also reported that myotomal weakness was associated with improvement in one of their chosen outcome measures (leg pain), but other studies report on neurologic deficits and their likely association with nonimprovement [13], [34]. The finding of initial myotomal weakness being associated with improvement may reflect the fact that the most common reason for nerve root compression causing sciatic pain and neurological deficits, is a disc prolapse, which often improves spontaneously leading to improvement in pain and disability. Another possibility is that prognostic factors may be different in primary care cohorts, such as ours, compared to secondary care cohorts [13].

### Strengths and limitations

As the majority of patients with back-related leg pain and sciatica are managed in primary care, one major strength of our study is the primary care setting, thereby providing important new evidence in a sample that is more representative of people with sciatica consulting healthcare than previously reported secondary care cohorts. The inclusion of consecutive eligible patients with any degree of pain severity and duration of symptoms further strengthens the generalizability of our findings, as these are applicable across the spectrum of patient presentations and not only for those populations with the most severe symptoms. The choice of potential prognostic factors was comprehensive and underpinned by previous research and clinical experience. The sensitivity analysis using the RMDQ continuous scales produced similar results to the primary analysis using the dichotomous outcome increasing confidence in the results.

A potential limitation is the higher than expected attrition; however, the multiple imputation sensitivity analysis showed similar estimates. Another limitation is that, because of small numbers in the referred leg pain subgroup, we were unable to do separate analyses as we did in the sciatica subgroup. We therefore cannot confirm that similar factors are associated with outcome in patients with referred leg pain.

Another issue is the potential confounding by treatment, where beneficial effects of treatment may influence the association between prognostic factors and outcome [35], [36]. To address this issue, we estimated the strength of association with and without adjustment for care pathways. As results remained broadly similar, we are reassured that treatment did not have a significant impact on our findings. A further limitation in terms of treatment may be the use of analgesic medication. Patients were treated as per usual practice as regards analgesia; however, we do not have data on this and therefore we are not able to adjust for or comment on the potential effect of analgesic use.

Lastly, it is important to consider the issue of uncertainty when diagnosing sciatica versus referred leg pain, and the potential for misclassification. In the absence of a “gold standard” for the diagnosis of sciatica, diagnosis in this study was based on clinical opinion based on the clinical assessment findings, which reflects normal primary care practice. Extensive discussion of these points is presented elsewhere [18]. However, the baseline (clinical examination) characteristics of the subgroup clinically diagnosed with sciatica are clearly different from those of the subgroup diagnosed with referred leg pain, and in line with the symptoms and signs expected to be present in the clinical diagnosis of sciatica, although the possibility of misclassification still remains. Furthermore, the sensitivity analysis using the subsample with clinical diagnosis of sciatica and corroborative MRI findings of nerve root compression found the same factor (identity) associated with outcome, which indicates that the influence of potential diagnostic misclassification on results does not appear to be significant.

### Suggestions for further research

Of the prognostic factors we investigated, independent predictors of improvement were similar in the whole sample and in the sciatica subgroup (which was the largest), with clinical characteristics more weakly associated with outcome and no longer significant in the combined model. This suggests that long-term outcome may be more strongly influenced by factors indicative of overall impact of the condition as indicated by the “identity” variable; therefore, this should influence early management and treatment intensity. Although we included most factors currently considered potentially important, it is possible that there are still unknown characteristics especially relevant to sciatica that we are not capturing, and which may better guide choice of intensity and timing of different care pathways. More specific MRI findings (eg, size of disc herniation) were not included as prognostic variables, mainly because of the primary care setting, which does not include routine MRI for this population. However, MRI characteristics can be investigated in further analysis to assess their contribution to the generic factors identified in this study.

We could not disentangle whether the prognostic factors mediate or moderate treatment effect. Further research investigating different models of care (eg, early and intensive interventions for patients with high overall impact of symptoms and a reduced expectation of a timely recovery versus current models of “stepped” care), and incorporating the prognostic factors this study identified, may elucidate which factors are prognostic and which are predictive of treatment outcome.

## Conclusions

At 1 year, 55.0% of primary care patients with low back-related leg pain and sciatica receiving current best care reported a 30% or more improvement in disability. In the long term, patients' belief that they will get better soon, and not having many other complaints attributed to the back and leg pain, were independent prognostic factors associated with improvement. These prognostic factors can be used to inform and direct management decisions about timing and intensity of available therapeutic options for symptoms relief, especially in sciatica patients with corroborative MRI findings, for whom there are potentially appropriate therapeutic interventions that are not applicable for patients with nonspecific low back and leg symptoms. Exploration and appropriate handling of patient's expectations about their pain trajectory, both in referred leg pain and in sciatica cases, is considered relevant, similarly to most health problems presentations.

## Supplementary material

Table S1 Baseline characteristics of participants followed up and lost to follow-up at 4 and 12 months. Table S1

Table S2 Multivariable associations between baseline characteristics and improvement in the RMDQ for the subsample of participants with sciatica and corroborative MRI findings of nerve root compression (n=252), combining all the six preselected set of variables. Table S2
