# Supplementary material for: Prognosis of sciatica and back-related leg pain in primary care: the ATLAS cohort
Source: Spine J. 2018 Jun;18(6):1030–40. doi: 10.1016/j.spinee.2017.10.071 (PMC5984249; doi:10.1016/j.spinee.2017.10.071)
Supplement: Table S1 — Baseline characteristics of participants followed up and lost to follow-up at 4 and 12 months. [file mmc1.docx]

Appendix A Table Baseline characteristics of participants followed-up and lost to follow-up at 4 and 12 months.

|  | **4 Months** | | **12 Months** | |
| --- | --- | --- | --- | --- |
| Key baseline characteristics | Followed up (n=402; 66%) | Lost to follow-up (n=207; 34%) | Followed up (n=450; 74%) | Lost to follow-up (n=159; 26%) |
|  |  |  |  |  |
| Age, Mean (SD) | 54.3 (13.0) | 42.3 (12.1) | 52.9 (13.2) | 42.5 (13.0) |
| Gender: Female, n (%) | 247 (61.4) | 136 (65.7) | 295 (65.6) | 88 (55.4) |
| Deprivation tertile, n (%) |  |  |  |  |
| Most deprived third | 111 (27.6) | 79 (38.5) | 134 (35.2) | 56 (35.2) |
| Middle | 134 (33.3) | 66 (32.2) | 146 (32.6) | 54 (34.0) |
| Least deprived third | 157 (39.1) | 60 (29.3) | 168 (37.5) | 49 (30.8) |
| RMDQ score, Mean (SD) | 12.1 (5.7) | 13.7 (12.9) | 12.1 (5.7) | 14.1 (5.5) |
| Leg pain intensity, Mean (SD) | 5.2 (2.4) | 5.2 (2.4) | 5.2 (2.4) | 5.3 (2.5) |
| Leg pain duration, n (%) |  |  |  |  |
| <6 weeks | 164 (42.5) | 87 (44.2) | 195 (45.1) | 56 (37.1) |
| 6 to 12 weeks | 82 (21.2) | 38 (19.3) | 86 (19.9) | 34 (22.5) |
| Over 3 months | 140 (36.3) | 72 (36.6) | 151 (35.0) | 61 (40.4) |
