# Supplementary material for: Prognosis of sciatica and back-related leg pain in primary care: the ATLAS cohort
Source: Spine J. 2018 Jun;18(6):1030–40. doi: 10.1016/j.spinee.2017.10.071 (PMC5984249; doi:10.1016/j.spinee.2017.10.071)
Supplement: Table S2 — Multivariable associations between baseline characteristics and improvement in the RMDQ for the subsample of participants with sciatica and corroborative MRI findings of nerve root compression (n=252), combining all the six preselected set of variables. [file mmc2.docx]

Appendix B Table. Multivariable associations between baseline characteristics and improvement in the RMDQ for the subsample of participants with sciatica and corroborative MRI findings of nerve root compression (n=252), combining all the six preselected set of variables.

|  | **12 months (n=201)** |
| --- | --- |
| **Variables in the final model (Reference category)** | **Adjusted for all the variables in the model, demographics & care pathways** |
|  | **OR (95% CI)** |
| **Sciatica with positive MRI findings** |  |
| Duration of leg pain (<6 weeks) |  |
| 6-12 weeks | 0.16 (0.02-1.16) |
| Over 3 months | 0.04 (0.004-0.39) |
| Timeline | 0.20 (0.04-1.00) |
| Identity | 0.41 (0.20-0.83) |
